# Bulk Ferromagnetic Icosahedral Quasicrystals without Rapid Quenching

**DOI:** 10.1021/jacs.6c03748

**Published:** 2026-07-07

**Authors:** Ryuji Tamura, Farid Labib, Kazuki Inagaki, Ryo Takeuchi, Takafumi Tsugawa, Takenori Fujii, Shintaro Suzuki, Asuka Ishikawa, Chang Liu, Minoru Kusaba, Ryo Yoshida

**Affiliations:** † Department of Materials Science and Technology, 378550 Tokyo University of Science, Tokyo 125-8585, Japan; ‡ Research Institute of Science and Technology, Tokyo University of Science, Tokyo 125-8585, Japan; § Cryogenic Research Center, 13143 The University of Tokyo, Bunkyo, Tokyo 113-0032, Japan; ∥ Department of Physical Science, Aoyama Gakuin University, Sagamihara, Kanagawa 252-0344, Japan; ⊥ School of Engineering, Institute of Engineering Innovation, 594905 The University of Tokyo, Tokyo 113-8656, Japan; # 170195 The Institute of Statistical Mathematics, Research Organization of Information and Systems, Tachikawa, Tokyo 190-8562, Japan; ∇ Advanced General Intelligence for Science Program (AGIS), TRIP Headquarters, RIKEN, Wako, Saitama 351-0198, Japan; ○ Fusion Science Interdisciplinary Coordination Center, 92172 National Institute for Fusion Science, Toki, Gifu 509-5292, Japan; ◆ Graduate University for Advanced Studies (SOKENDAI), Tachikawa, Tokyo 190-8562, Japan

## Abstract

While ferromagnetism is well understood in periodic and amorphous materials, its critical behavior in quasiperiodic systems has remained elusive. Although ferromagnetic icosahedral quasicrystals (iQCs) have recently been discovered, all known examples have been limited to rapidly quenched, metastable states, precluding annealing, structural refinement, and quantitative studies of magnetic criticality. Here we demonstrate, for the first time, the realization of bulk, annealable ferromagnetic iQCs in compositionally tuned Au–Cu–Al–In–R (R = Gd, Tb, Dy) alloys. Guided by chemical design in multicomponent alloy space, single-phase iQCs are obtained by conventional arc melting and subsequent controlled annealing, resulting in sharp quasiperiodic diffraction and exceptional thermal robustness. Strikingly, while all compounds exhibit clear ferromagnetic order, their magnetic critical behavior differs systematically depending on the rare-earth element. Tb- and Dy-based iQCs display critical exponents close to mean-field values, whereas the Gd-based system exhibits a significantly enhanced critical exponent δ, deviating from mean-field behavior and from both anisotropic counterparts and previously studied approximant crystals. This difference is attributed to stronger spin fluctuations in the isotropic Gd system, which effectively shorten the interaction range, while anisotropy in Tb/Dy suppresses fluctuations and leads to mean-field-like behavior. These results establish that magnetic criticality in quasicrystals is not uniquely determined by quasiperiodic order alone but is governed by the interplay between quasiperiodicity and local spin symmetry. More broadly, they demonstrate that quasiperiodic solids provide a tunable materials platform for accessing nonmean-field critical behavior beyond the constraints of periodic systems.

## Graphical abstract

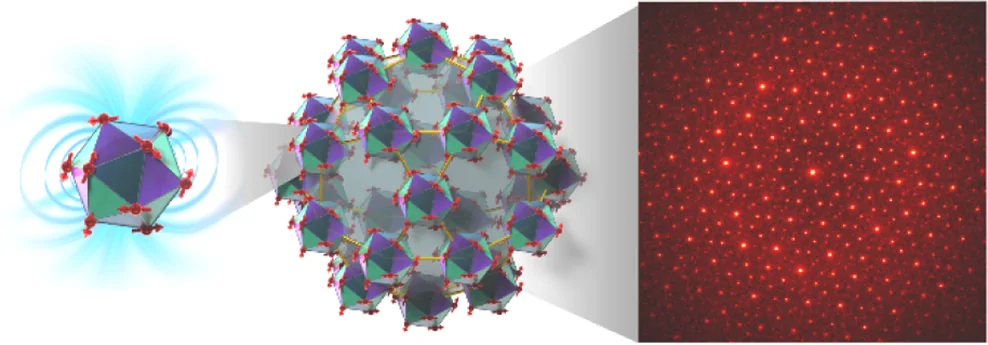

## Introduction

Ferromagnetism, characterized by spontaneous magnetization, has been extensively studied in periodic crystals^1,2^ and amorphous materials,^3^ establishing a broad framework for understanding magnetic order in systems with and without translational symmetry, respectively. In contrast, quasicrystals (QCs), which possess long-range quasiperiodic order and noncrystallographic rotational symmetries,^4,5^ have long posed a fundamental challenge to this framework. Despite intensive efforts since their discovery, magnetic QCs were long believed to support only short-range or glassy magnetic states, with no experimental evidence for long-range ferromagnetic order.^6–9^

This situation changed with the discovery of ferromagnetic and antiferromagnetic icosahedral quasicrystals (iQCs) in Au-based alloys,^10–12^ demonstrating that spontaneous magnetization and long-range magnetic order can emerge even on a quasiperiodic lattice. These findings positioned QCs as a third platform for studying magnetic order, distinct from both periodic crystals and amorphous systems. However, a critical limitation has persisted: all ferromagnetic QCs reported so far can only be obtained by rapid quenching, rendering them metastable and structurally imperfect. Upon annealing, they readily transform into periodic approximant or crystalline phases, preventing improvement of quasiperiodic order and severely restricting quantitative investigations of intrinsic magnetic properties.

A slab packing of rhombic triacontahedron clusters in three-dimensional physical space based on the prototype Tsai-type Cd–Yb QC model is illustrated in Figure 1a. Each rhombic triacontahedron consists of concentric inner shells: an icosidodecahedron (30 atoms), a rare-earth (R) icosahedron (12 atoms), a dodecahedron (20 atoms), and a central tetrahedron (Figure 1b). The R atoms occupy two crystallographically distinct sites: the 12 vertices of the icosahedron and two additional sites located along the body-diagonal axis of the acute rhombohedron unit, which fills the interstitial space between neighboring rhombic triacontahedron clusters (see ref 13 for structural details). The quasiperiodic magnetic lattice formed by the R moments, viewed along a 5-fold symmetry axis, is shown in Figure 1c. This magnetic lattice fundamentally differs from both uniform periodic lattices and random amorphous networks, and is therefore expected to play a decisive role in shaping magnetic fluctuations near criticality. Nevertheless, the absence of bulk, annealable ferromagnetic QCs has so far precluded reliable determination of magnetic critical behavior on a quasiperiodic lattice.

**Figure 1:**
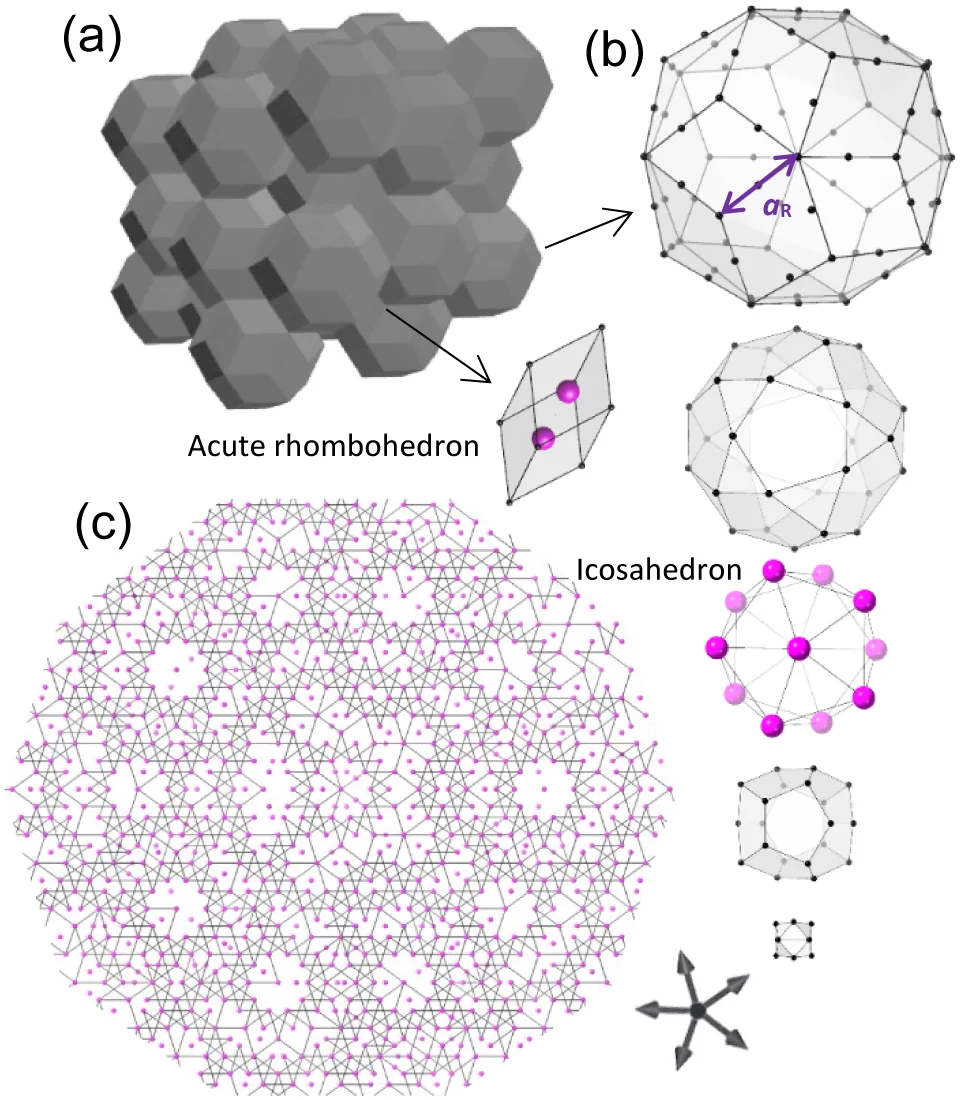
(a) A slab packing of rhombic triacontahedron clusters in three-dimensional physical space based on the Tsai-type Cd–Yb QC model. (b) Shell structure of the rhombic triacontahedron encompassing four inner shells; from the outermost shell to the center: a rhombic triacontahedron with 92 atoms sites, an icosidodecahedron (30 atomic sites), an icosahedron (12 atomic sites), a dodecahedron (20 atomic sites) and an orientationally disordered innermost tetrahedron (4 atomic sites). The edge length of the rhombic triacontahedron’s surface rhombi is denoted by *a*_R_. The six-dimensional quasilattice parameter *a*_6D_ (see the text for the details) equals to $a_{R}\times \sqrt{2}$ . The rare earth (R) atoms (represented by pink atoms) occupy two crystallographically distinct sites: (i) the 12 vertices of the icosahedron and (ii) two sites located along the body-diagonal axis of the acute rhombohedron unit that fills the interstitial space between neighboring rhombic triacontahedron units. (c) Quasiperiodic magnetic lattice formed by R moments in an icosahedral quasicrystal (iQC), viewed along a 5-fold symmetry axis.

As a result, a central question in QC magnetism has remained unanswered: how magnetic criticality manifests when high quasiperiodic structural order is achieved, and whether it depends on microscopic magnetic degrees of freedom. Addressing this question requires bulk ferromagnetic QCs with sufficient structural coherence to enable quantitative evaluation of critical exponents—an experimental regime that has remained inaccessible.

In this work, we achieve two key advances. First, we realize bulk, annealable ferromagnetic iQCs without rapid quenching by means of compositionally tuned multicomponent alloying guided by a machine-learning-based phase classifier. Second, this unprecedented sample quality enables quantitative determination of magnetic critical behavior. Critical exponents describe how physical quantities evolve near continuous phase transitions and provide insight into the nature and effective range of the underlying magnetic interactions.^14^ In QCs, the absence of translational periodicity raises the question of whether their critical behavior follows well-established universality classes such as the mean-field model,^14^ or instead requires new theoretical frameworks for its description.

Analysis of the critical exponents in the present iQCs reveals an unexpected difference between the Tb- and Dy-based (non-Heisenberg) systems and their Gd-based (Heisenberg) counterpart on the same quasiperiodic lattice. The Tb- and Dy-based iQCs exhibit exponents close to mean-field values, whereas the Gd-based iQC shows a significantly enhanced δ, indicating a clear deviation from mean-field behavior. These results are discussed in terms of the effective range of long-range magnetic interactions within theoretical frameworks developed elsewhere.^15,16^

## Results

### Machine Learning

A systematic analysis of the approximant crystals dataset revealed that ferromagnetic ordering tends to emerge at a electron-per-atom ( *e/a*) of ≈ 1.7 ^17,38^ In this study, starting from the previously reported metastable ternary ferromagnetic QCs in Au_65_Ga_20_R_15_ (R = Gd, Tb, Dy) iQCs alloy systems, we explored stable quinary ferromagnetic QCs while maintaining *e/a* ≈ 1.7.

Using the QC database HYPOD-X^18^ and other existing databases, we constructed a five-class classifier to predict whether a given composition forms an iQC, icosahedral approximant crystal, decagonal QC, decagonal approximant crystal, or other phases (including conventional crystals) (see Experiments). To generate candidate compositions, we selected two elements for the Au site from {Au, Pd, Cu, Ag, Cd, Ni}, two elements for the SM site from {Ga, B, Al, In, Si, Ge}, and one element for the R site from {Gd, Tb, Dy}, starting from Au_65_SM_20_R_15_, thereby generating 675 quinary alloy systems satisfying 1.65 ≤ *e/a* ≤ 1.75.

For each system, we evaluated the class probability for iQC formation and ranked the candidates accordingly. The Au–Cu–Al–In–R (R = Gd, Tb, Dy) systems were found among the highest-ranked candidates (first, 16th, and 21st, respectively), which led to the successful synthesis of three new bulk quinary ferromagnetic QCs discussed in the present study.

### Materials Design Strategy and Structural Verification

We demonstrate that high-quality, thermally robust bulk iQCs can be realized in Au–Cu–Al–In–R (R = Gd, Tb, Dy) systems, overcoming the long-standing limitation of metastability in previously reported ferromagnetic iQCs. These iQCs provide reliable platform for investigating intrinsic magnetic criticality in quasiperiodic lattices.

Figure 2 shows the powder X-ray diffraction patterns of the synthesized iQCs. All observed diffraction peaks are consistently indexed as primitive iQCs using Elser’s indexing scheme^19^ (see Experiments), confirming the formation of single-phase iQCs. Notably, in Figure 2, the diffraction peaks are significantly sharper than those reported for ternary ferromagnetic iQCs obtained by rapid quenching,^10,11^ evidencing a substantial enhancement of quasiperiodic order achieved through multicomponent alloying.

**Figure 2:**
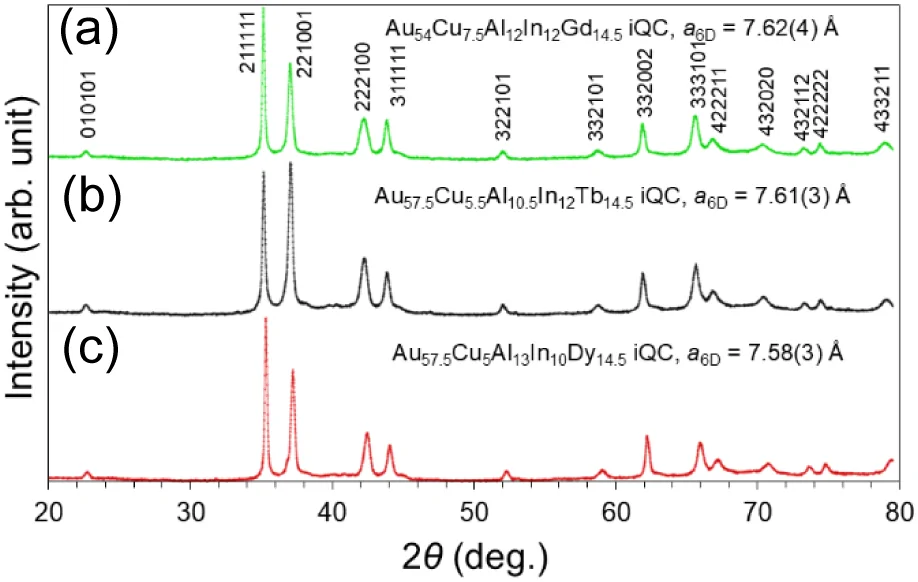
Powder X-ray diffraction patterns of annealed (a) Au_54_Cu_7.5_Al_12_In_12_Gd_14.5_, (b) Au_57.5_Cu_5.5_Al_10.5_In_12_Tb_14.5_, and (c) Au_57.5_Cu_5_Al_13_In_10_Dy_14.5_ alloys synthesized by conventional arc melting followed by controlled annealing. All reflections are consistently indexed as primitive icosahedral QCs using Elser’s indexing scheme, demonstrating single-phase iQC formation in bulk samples without rapid quenching. The diffraction peaks are markedly sharper than those of previously reported rapidly quenched ferromagnetic iQCs, evidencing substantially improved quasiperiodic order enabled by multicomponent alloying and annealing, which is essential for quantitative investigations of intrinsic magnetic critical behavior. Weak shoulders around 2θ ≈ 37°, 38°, and 45° in the Tb- and Dy-based QCs have less than ∼4% integrated intensities of the [221001] Bragg reflection and cannot be indexed as a separate crystalline phase. These shoulders are likely originate from minor compositional inhomogeneity or local structural variations in the multicomponent alloy.

The nominal compositions Au_54_Cu_7.5_Al_12_In_12_Gd_14.5_, Au_57.5_Cu_5.5_Al_10.5_In_12_Tb_14.5_ and Au_57.5_Cu_5_Al_13_In_10_Dy_14.5_ correspond to valence-electron concentrations *e/a* = 1.77, 1.74, and 1.75, respectively, close to the empirical optimum for ferromagnetism in Tsai-type systems (*e/a* ≈ 1.7).^17,38,20^

These quinary iQCs remain stable under prolonged annealing at 723 K and below, in contrast to their transformation into approximant crystals (i.e., periodic structures that share identical local atomic clusters as the parent QC but are arranged in a periodic lattice^13^) at higher temperatures, showing exceptionally high thermal stability. Longer heat treatments at 723 K further sharpen the diffraction peaks, indicating enhanced long-range quasiperiodic coherence rather than structural degradation. The averaged 6-dimensional lattice parameters (*a*_6D_), extracted from the (211111) and (332002) reflections, are 7.62(4) Å, 7.61(3) Å, and 7.58(3) Å for the Gd-, Tb- and Dy-based iQCs, respectively. These values are consistent with Tsai-type iQCs, where $a_{6D}=\sqrt{2}a_{R}$ , with *a*_R_, quasilattice parameter, corresponding to the edge length of the rhombic triacontahedral structural unit (see Figure 1b). Selected-area electron diffraction patterns (Figure 3) taken with indices along 5-fold, 3-fold, and 2-fold symmetry axes further confirm the high structural quality of the primitive iQCs. The sharp reflections and the presence of numerous weak spots—absent in rapidly quenched ternary iQCs^10,11^ —indicate a significantly enhanced degree of quasiperiodic order. These results establish that metastable ternary ferromagnetic iQCs can be transformed into bulk, annealable, and structurally well-defined QCs through multicomponent isovalent substitution. Having established this materials platform, we next examine whether long-range magnetic order can emerge in these quasiperiodic solids.

**Figure 3:**
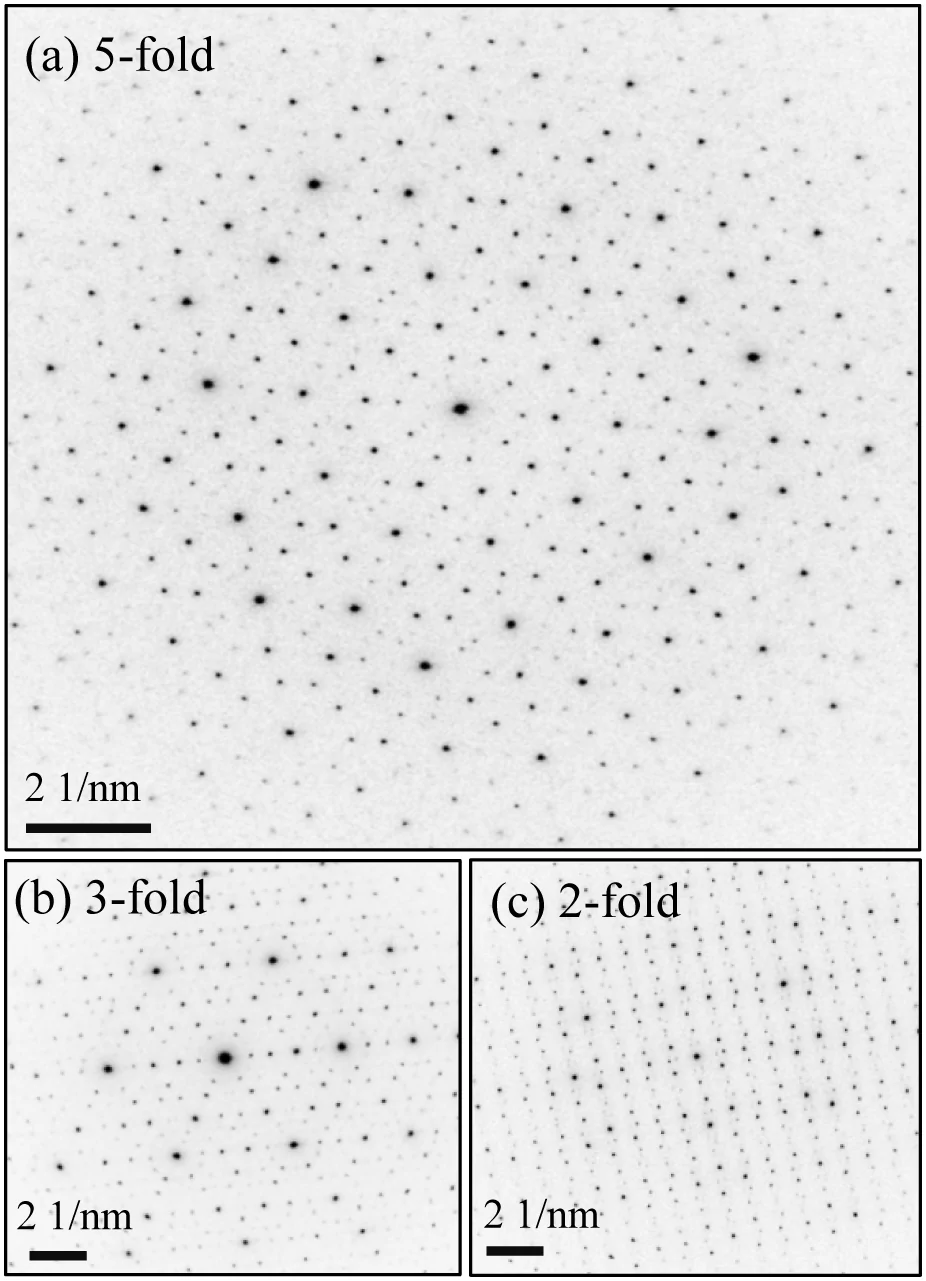
Selected-area electron diffraction patterns of the annealed Au_57.5_Cu_5.5_Al_10.5_In_12_Tb_14.5_ iQC taken with indices along (a) 5-fold, (b) 3-fold and (c) 2-fold symmetry axes. Sharp fundamental reflections accompanied by numerous weak spots demonstrate a high degree of long-range quasiperiodic coherence in the bulk state. Compared with previously reported rapidly quenched ferromagnetic iQCs, the present patterns exhibit significantly enhanced coherence, directly confirming that multicomponent isovalent substitution stabilizes long-range quasiperiodic order in bulk samples without rapid quenching.

### Establishment of Bulk Ferromagnetic Order in Icosahedral Quasicrystals

We establish bulk long-range ferromagnetic order in Gd, Tb, and Dy-based iQCs, providing clear evidence that quasiperiodic systems can support intrinsic ferromagnetism. Figure 4 shows the temperature dependence of the dc magnetic susceptibility for the Gd-, Tb- and Dy-based iQCs. All samples exhibit clear divergences indicative of ferromagnetic transitions at *T*_C_ = 28.3, 16.5, and 9.7 K, respectively. The inverse susceptibility (*H/M*) measured under 1000 Oe (inset of Figure 4) follows a nearly linear trend fitting well to the Curie–Weiss law: $\chi \left(T\right)=N_{A}\mu_{\mathrm{eff}}^{2}\mu_{B}^{2}/3k_{B}\left(T-\theta_{w}\right)+\chi_{0}$ , where *N*_A_, μ_eff_, μ_B_, *k*_B_, θ_w_, and χ_0_ denote the Avogadro number, effective magnetic moment, Bohr magneton, Boltzmann constant, Curie–Weiss temperature, and the temperature-independent magnetic susceptibility, respectively. Linear least-squares fitting across 50–300 K yields positive θ_w_ values of 26.67(5), 13.18(4), and 7.84(4) K for the Gd-, Tb-, and Dy-based iQCs, respectively, indicating dominant ferromagnetic interactions. The extracted effective magnetic moments are in close agreement with the free-ion values for Gd^3+^ (8.339(4)­μ_B_), Tb^3+^ (10.083(3)­μ_B_), and Dy^3+^ (10.994(3)­μ_B_),^21^ confirming well-localized magnetic moments on the rare earth ions.

**Figure 4:**
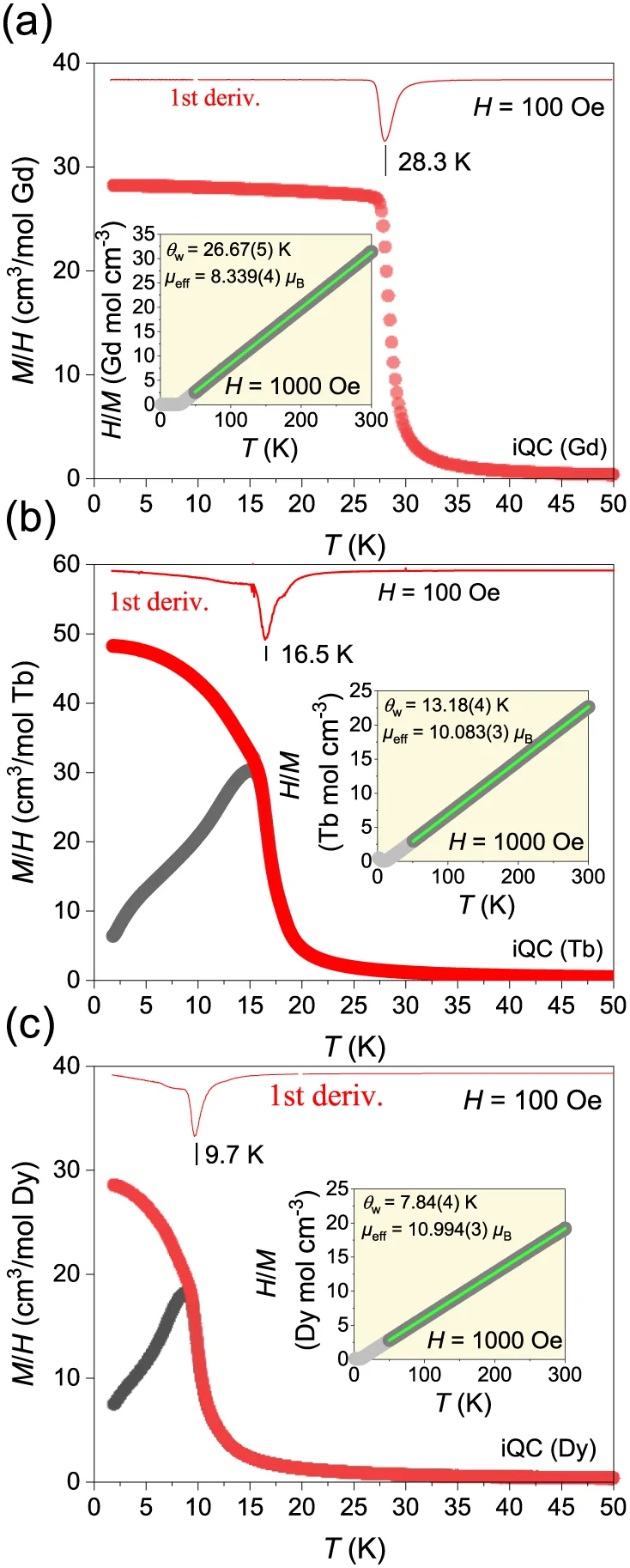
Temperature dependence of *M*/*H* for (a) Au_54_Cu_7.5_Al_12_In_12_Gd_14.5_, (b) Au_57.5_Cu_5.5_Al_10.5_In_12_Tb_14.5_, and (c) Au_57.5_Cu_5_Al_13_In_10_Dy_14.5_ iQCs measured under zero-field-cooled (ZFC, gray) and field-cooled (FC, red) conditions. Clear divergences indicate ferromagnetic transitions. Insets show the inverse susceptibility *H/M* over 1.8–300 K, revealing Curie–Weiss behavior with positive Weiss temperatures. Fits to the Curie–Weiss law yield temperature-independent terms *χ*_0_ = 1.18(2) × 10^–4^, 2.31(2) × 10^–4^, and 4.15(3) × 10–^4^ cm^3^.mol^–1^ for the Gd-, Tb-, and Dy-based iQCs, respectively.

Specific heat measurements (Figure 5a) reveal pronounced λ-type anomalies at the corresponding transition temperatures, unambiguously confirming long-range ferromagnetic order. Compared with earlier Gd-based ferromagnetic QCs obtained by rapid quenching,^10,11^ the anomalies are significantly sharper, reflecting the improved structural quality of the present bulk samples. Moreover, the in-phase component of the AC magnetic susceptibility in the Gd-based QC (see Figure S1) shows no frequency dependence, consistent with conventional long-range ferromagnetism. The Tb- and Dy-based QCs exhibit only small peak shifts of ∼0.02 K and ∼0.04 K per decade of frequency, respectively. These correspond to Mydosh parameters (defined as δ = Δ*T*_*f*_/[*T_f_* Δlog­(*f*)])^22^ of 0.001 and 0.003, respectively, which are significantly smaller than those of canonical spin-glass systems (typically δ ≈ 0.01–0.05),^22–25^ thereby ruling out spin-glass freezing behavior in these samples. The weak frequency dependence likely reflects slow spin relaxation associated with strong single-ion anisotropy rather than glassy behavior.

**Figure 5:**
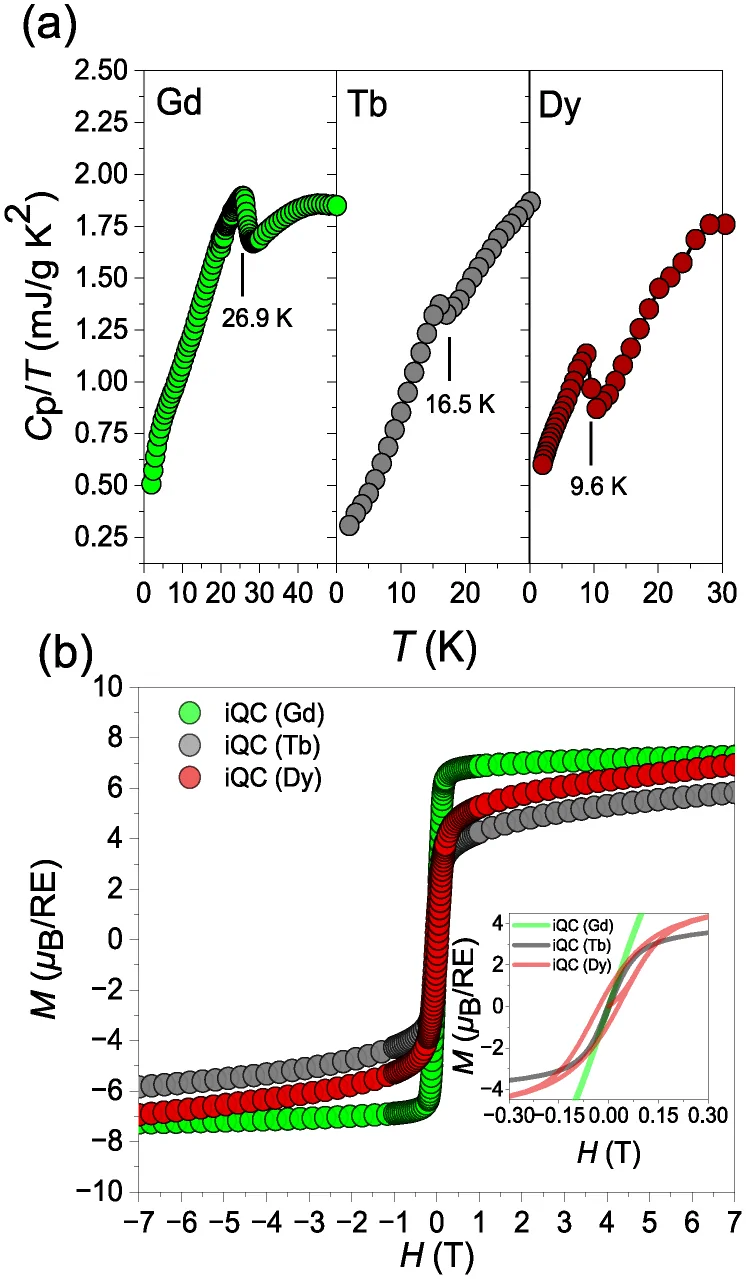
(a) Temperature dependence of *C*_p_/*T* for Au_54_Cu_7.5_Al_12_In_12_Gd_14.5_, Au_57.5_Cu_5.5_Al_10.5_In_12_Tb_14.5_, and Au_57.5_Cu_5_Al_13_In_10_Dy_14.5_ iQCs. All compounds exhibit pronounced λ-type anomalies at their respective Curie temperatures, evidencing bulk long-range ferromagnetic order. (b) Field-dependent magnetization measured at 1.8 K up to μ_0_ *H* = 7 T. The Gd-based iQC rapidly saturates to the full Hund’s-rule moment of 7.0 μ_B_/Gd^3+^, whereas the Tb- and Dy-based iQCs remain unsaturated due to strong single-ion anisotropy.

Field-dependent magnetization curves (Figure 5b) show rapid saturation of the Gd-based iQC to the full Hund’s-rule moment of 7.0 μ_B_/Gd^3+^ at low field (∼0.6 T), consistent with isotropic Heisenberg behavior. In contrast, the Tb- and Dy-based iQCs do not reach full saturation up to 7 T, attaining only about two-thirds of their respective free-ion moments (9.0 μ_B_/Tb^3+^ and 10.0 μ_B_/Dy^3+^). This behavior is characteristic of non-Heisenberg QCs^10,11^ and is attributed to strong single-ion anisotropy associated with nonzero orbital angular momentum of Tb^3+^ and Dy^3+^, as described elsewhere.^26,27^ These results demonstrate that structurally well-defined bulk iQCs host intrinsic long-range ferromagnetic order, rather than spin-glass-like freezing, establishing a new class of quasiperiodic ferromagnets. We now proceed to a quantitative analysis of their critical behavior and universality.

### Critical Behavior and Universality in Quasiperiodic Ferromagnets

We reveal that magnetic criticality in iQCs is not universal but strongly element dependent, even within an identical quasiperiodic lattice. The high structural quality of the present iQCs enables a quantitative determination of intrinsic magnetic critical behavior. Standard Arrott plots (*H*/*M* versus *M* ^2^, Figure S2) show no negative slopes or inflection points, confirming second order ferromagnetic transitions according to the Banerjee criterion.^28^ This allows reliable scaling analysis based on established critical relations. For a second-order transition, the spontaneous magnetization below *T*_C_ and the initial inverse susceptibility above *T*_C_ follow the scaling relations:^14^

1 $$M_{s}\left(T\right)=M_{0}{\left(-\epsilon \right)}^{\beta };\;\epsilon <0;\;T<T_{c}$$

2 $${\left(H/M\right)}_{0}\left(T\right)=\left(h_{0}/M_{0}\right)\epsilon^{\gamma };\;\epsilon >0;\;T>T_{c}$$

where *M*_0_ and *h*_0_ denote the critical amplitudes while ϵ = (*T – T*_C_)/*T*_C_ represents the reduced temperature. Following the framework described elsewhere,^29^ we obtain (β, γ) = (0.41,1.17), (0.51, 0.91), and (0.47, 0.89) for the Gd-, Tb-, and Dy-based iQCs, respectively (Table 1). Modified Arrott plots constructed with these exponents exhibit excellent linearity near *T*_C_ (Figure 6 and Figure S3), demonstrating internal consistency.

**Table 1:** Critical Exponents β, γ, and δ Obtained for the Present Bulk iQCs are Compared with Representative Theoretical Universality Classes^a^

System/Universality class	β	γ	δ	*T*_C_ (K)
Au_54_Cu_7.5_Al_12_In_12_Gd_14.5_ iQC	0.41	1.17	3.85	26.9
Au_57.5_Cu_5.5_Al_10.5_In_12_Tb_14.5_ iQC	0.51	0.91	2.78	16.5
Au_57.5_Cu_5_Al_13_In_10_Dy_14.5_ iQC	0.47	0.89	2.89	9.6
Au_65_Ga_20_Dy_15_ iQC^10^	0.54	0.89	2.66	7.5
Icosahedral Heisenberg (MC)^15^	0.508	1.361	3.68	–
Mean-field	0.50	1.00	3.00	–
Tricritical Mean-Field	0.25	1.00	5.00	–
3D Ising	0.325	1.24	4.82	–
3D Heisenberg	0.365	1.386	4.82	–

a The approximate uncertainties in the critical exponents are ±0.03 for β, ±0.07 for γ, and ±0.18 of δ. The Gd-based iQC exhibits a set of critical exponents that deviate from those of conventional universality classes, including mean-field, tri-critical mean-field, and three-dimensional Ising or Heisenberg models.

**Figure 6:**
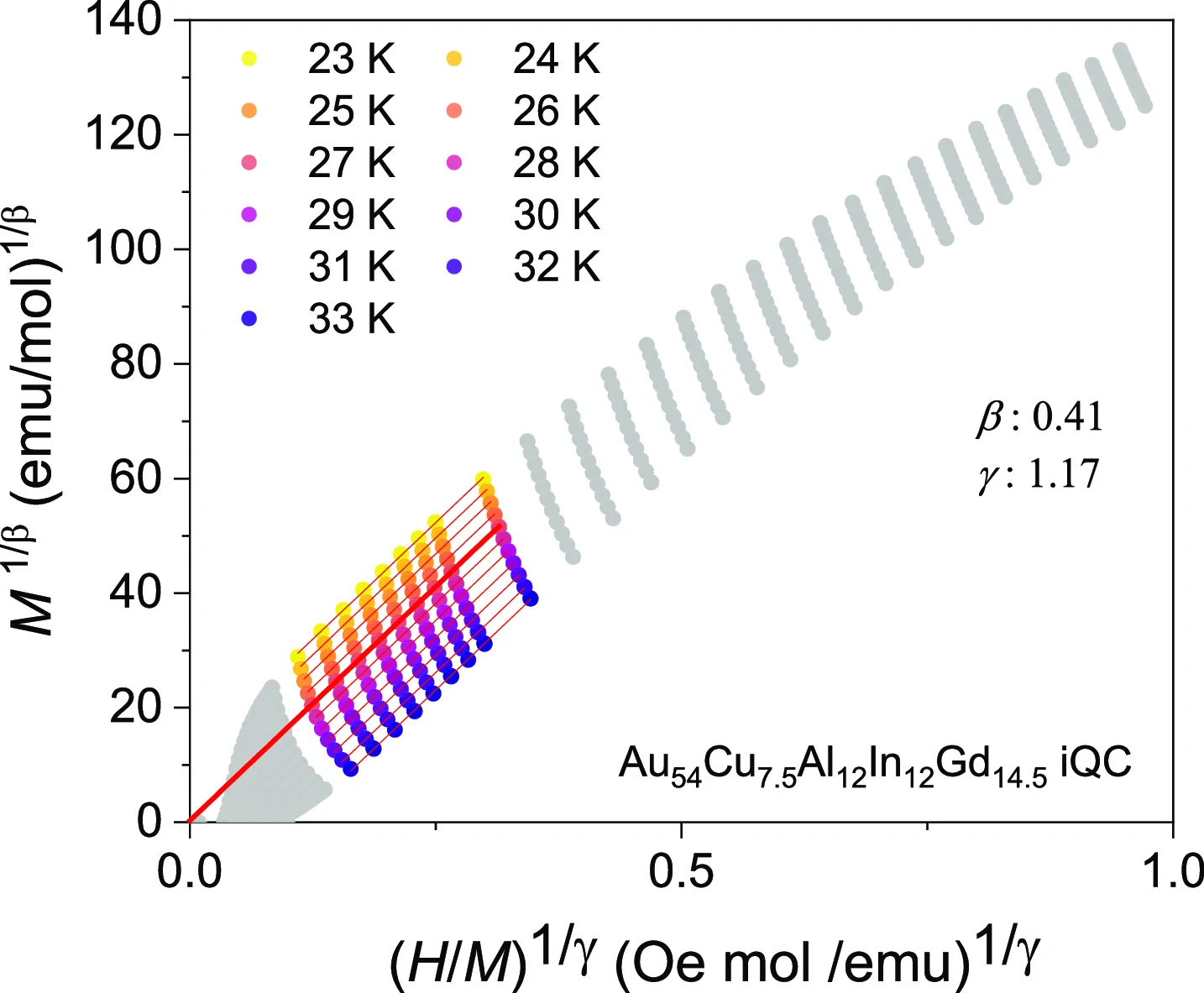
Modified Arrott isotherms plotted as *M* ^1/β^ versus *H/M* ^1/γ^ for the Au_54_Cu_7.5_Al_12_In_12_Gd_14.5_ iQC. The nearly parallel and linear behavior of the isotherms in the highlighted field range (0.4 *T* < *H* < 2.5 T) demonstrates the self-consistency of the critical exponents and confirms a second-order ferromagnetic phase transition.

Applying Widom’s relation δ = 1 + γ/β^30^ yields δ = 3.85, 2.78, and 2.89 for the Gd-, Tb-, and Dy-based iQCs, respectively. Despite sharing an identical quasiperiodic lattice, these compounds exhibit markedly different critical behaviors. The Tb- and Dy-based iQCs show exponents close to mean-field values,^31^ indicating essentially mean-field-like ferromagnetic transitions. In contrast, the Gd-based iQC exhibits reduced β and enhanced γ, resulting in a larger δ and clear deviation from mean-field behavior. This contrast can be understood in terms of the effective interaction range. As the effective interaction range becomes shorter, deviations from mean-field exponents naturally emerge.^16^ In particular, a reduction in β and an increase in γ naturally emerge as the system moves away from the infinite-interaction-range limit characteristic of the mean-field regime. Although the exact form of the RKKY interaction in quasicrystals remains an open question, the observed trend in the Gd-based system is qualitatively consistent with this picture.

A microscopic origin of this difference lies in spin symmetry. The isotropic Gd^3+^ ion (*L* = 0) allows strong spin fluctuations, effectively renormalizing the interaction range. In contrast, Tb^3+^ and Dy^3+^ exhibit strong single-ion anisotropy due to finite orbital angular momentum, suppressing spin fluctuations and stabilizing mean-field-like behavior.

These results establish that magnetic criticality in QCs is governed not solely by quasiperiodicity, but by the interplay between quasiperiodic geometry and local spin symmetry. Quasiperiodicity thus acts as an active degree of freedom that enables access to unconventional, nonmean-field critical behavior when combined with appropriate microscopic interactions.

## Conclusions

We establish the first bulk, annealable ferromagnetic icosahedral quasicrystals, overcoming the long-standing limitation of metastable, rapidly quenched states and enabling access to intrinsic quasiperiodic magnetism.

Despite sharing an identical icosahedral quasiperiodic framework, the present quasicrystals exhibit markedly different magnetic critical behavior depending on the rare-earth element. Tb- and Dy-based quasicrystals display essentially mean-field-like criticality, whereas the Gd-based system exhibits a significantly enhanced critical exponent δ, deviating from the mean-field regime. This difference is attributed to stronger spin fluctuations in the isotropic Gd system, which effectively shorten the interaction range, while anisotropy in Tb/Dy suppresses fluctuations and leads to mean-field-like behavior.

These findings demonstrate that magnetic criticality in quasicrystals is not uniquely determined by quasiperiodic order alone, but emerges from a subtle interplay between quasiperiodic geometry and local spin symmetry. More broadly, the realization of bulk, annealable ferromagnetic quasicrystals establishes quasiperiodic solids as a robust materials platform for experimentally accessing nonmean-field critical behavior beyond the constraints of periodic lattices.

## Experiments

Polycrystalline samples with nominal compositions Au_54_Cu_7.5_Al_12_In_12_Gd_14.5_, Au_57.5_Cu_5.5_Al_10.5_In_12_Tb_14.5_, and Au_57.5_Cu_5_Al_13_In_10_Dy_14.5_ were synthesized by conventional arc melting under an argon atmosphere. Each ingot was remelted several times to ensure chemical homogeneity and subsequently annealed isothermally at *T* = 723 K for 2 weeks. Phase identification was performed by powder X-ray diffraction (PXRD) using a Rigaku SmartLab SE diffractometer with Cu–Kα radiation. The diffraction peaks of QCs are indexed using Elser’s indexing scheme,^19^ in which the Bragg peaks are described as projections of reciprocal lattice points from a six-dimensional periodic lattice onto physical three-dimensional space. In this framework, the diffraction pattern of an icosahedral QC is generated by six basis vectors, and each peak can be indexed by six integers corresponding to reciprocal lattice points in the higher-dimensional lattice. Selected-area electron diffraction measurements were carried out for a representative sample with nominal composition Au_57.5_Cu_5.5_Al_10.5_In_12_Tb_14.5_ using a JEM-2010F transmission electron microscope at the Advanced Research Infrastructure for Materials and Nanotechnology (ARIM), the University of Tokyo. DC magnetic susceptibility measurements under zero-field-cooled (ZFC) and field-cooled (FC) conditions were performed using a superconducting quantum interference device (SQUID) magnetometer (Quantum Design MPMS3) over the temperature range 1.8–300 K under external magnetic fields up to 7 T. Specific heat measurements were carried out using a Physical Property Measurement System (Quantum Design PPMS) at the Cryogenic Center at the University of Tokyo.

A machine-learning-based phase classifier was developed to predict QC formation from chemical composition alone. Chemical compositions were converted into 232-dimensional descriptor vectors using the Python library XenonPy.^33,34^ These descriptors were used to train a random forest classifier implemented in `scikit-learǹ,^35^ which outputs classification probabilities for five classes: icosahedral quasicrystal (iQC), icosahedral approximant crystal (iAC), decagonal quasicrystal (dQC), decagonal approximant crystal (dAC), and others (conventional crystalline phases). The training data set consisted of experimentally reported quasicrystals and approximant crystals compiled in the HYPOD-X database, augmented with 41 additional data sets accumulated in our laboratory. The number of samples in each class was 336 (iQC), 321 (iAC), 15 (dQC), and 13 (dAC). For conventional crystalline phases, 48,638 compositions were randomly selected from the Materials Project^36^ and Starrydata databases.^37^ The dataset was randomly partitioned into 70% for training, 15% for validation, and 15% for testing. Hyperparameters of the random forest classifier, including `n_estimators̀, `max_depth̀, and `bootstrap̀, were optimized using the validation set. Notably, only three quinary approximant-crystal compositions were included in the training data set, and no quinary quasicrystal compositions were used. Most quinary compositions were drawn from the Materials Project and Starrydata database, none of which contained Au. Consequently, the present study explored a markedly extrapolative regime of chemical composition space.

## Supplementary Material



## Notes

Frequency dependence of the ac magnetic susceptibility for Gd-, Tb-, and Dy-based iQCs; conventional and modified Arrott plot analyses; verification of second-order ferromagnetic phase transitions using the Banerjee criterion; and supporting critical-scaling analyses (PDF)
