# Supplementary material for: Bulk Ferromagnetic Icosahedral Quasicrystals without Rapid Quenching
Source: J Am Chem Soc. 2026 Jul 7;148(32):34154–60. doi: 10.1021/jacs.6c03748 (PMC13495759; doi:10.1021/jacs.6c03748)
Supplement: Supplementary file 1 [file ja6c03748_si_001.pdf]

Supporting Information

Bulk Ferromagnetic Icosahedral Quasicrystals without Rapid Quenching

Ryuji Tamura[1*], Farid Labib[2*], Kazuki Inagaki[1], Ryo Takeuchi[1], Takafumi Tsugawa[1], Takenori Fujii[3], Shintaro Suzuki[4], Asuka Ishikawa[5], Chang Liu[6,7], Minoru Kusaba[8], Ryo Yoshida[6, 7,9]

[1] *Department of Materials Science and Technology, Tokyo University of Science, Tokyo 125-8585, Japan*

[2] *Research Institute of Science and Technology, Tokyo University of Science, Tokyo 125-8585, Japan*

[3] *Cryogenic Research Center, The University of Tokyo, Bunkyo, Tokyo 113-0032, Japan*

[4] *Department of Physical Science, Aoyama Gakuin University, Kanagawa, Japan*

[5] *School of Engineering, Institute of Engineering Innovation, The University of Tokyo, Tokyo 113-8656, Japan*

[6] *The Institute of Statistical Mathematics, Research Organization of Information and Systems, Tachikawa, Tokyo 190-8562, Japan*

[7] *Advanced General Intelligence for Science Program (AGIS), TRIP Headquarters, RIKEN, Wako, Saitama 351-0198, Japan*

[8] *Fusion Science Interdisciplinary Coordination Center, National Institute for Fusion Science, Toki, Gifu 509-5292, Japan*

[9] *Graduate University for Advanced Studies (SOKENDAI), Tachikawa, Tokyo 190-8562, Japan*

*These authors contributed equally.

.

Critical Behavior Analysis

To establish the order of the ferromagnetic phase transitions and to validate the critical scaling analysis presented in the main text, we performed both conventional and modified Arrott plot analyses for all compositions.

Figure S1 shows the in-phase component of the ac magnetic susceptibility (χ'_{ac}) for (a) $Au_{54}Cu_{7.5}Al_{12}In_{12}Gd_{14.5}$, (b) $Au_{57.5}Cu_{5.5}Al_{10.5}In_{12}Tb_{14.5}$, and (c) $Au_{57.5}Cu_{5}Al_{13}In_{10}Dy_{14.5}$ measured over frequencies spanning two orders of magnitude (0.1–10 Hz). The Gd-based system exhibits essentially no frequency dependence, consistent with conventional long-range ferromagnetic ordering. In contrast, the Tb- and Dy-based systems show a weak frequency dependence in the magnitude of χ' below T_c. However, the peak temperature displays negligible shift with frequency, as shown in the inset of Fig. S1. To determine the peak temperature precisely, the data near the peak were fitted using a fourth-order polynomial function, $y = A_0 + A_1x + A_2x^2 + A_3x^3 + A_4x^4$. The resulting shift in peak temperature per decade of frequency is only 0.02 K for the Tb-based quasicrystal and 0.04 K for the Dy-based system. To quantify this behavior, we evaluated the Mydosh parameter, defined as $\delta = \Delta T_f/[T_f \Delta\log(f)]$. The obtained values are 0.001 for the Tb-based iQC and 0.003 for the Dy-based iQC. These values are more than an order of magnitude smaller than those of canonical spin-glass systems (e.g., $\delta \approx$ 0.01–0.05), demonstrating that the observed behavior is inconsistent with spin-glass freezing. Importantly, no frequency-dependent shift characteristic of spin-glass transitions is observed.

Figure S2 shows the conventional Arrott plots in the form of H/M versus M^2 for (a) $Au_{54}Cu_{7.5}Al_{12}In_{12}Gd_{14.5}$, (b) $Au_{57.5}Cu_{5.5}Al_{10.5}In_{12}Tb_{14.5}$, and (c) $Au_{57.5}Cu_{5}Al_{13}In_{10}Dy_{14.5}$ iQCs. For all three compounds, the isotherms exhibit neither negative slopes nor inflection points in the vicinity of Tc, satisfying the Banerjee criterion and confirming that the ferromagnetic transitions are continuous and of second order.

Figure S3 presents the modified Arrott isotherms plotted as $M^{1/\beta}$ versus $(H/M)^{1/\gamma}$, using the critical exponents determined self-consistently as described in the main text. For the Tb- and Dy-based iQCs, the isotherms display nearly parallel linear behavior over a well-defined field range (0.4 T < H < 2.5 T), demonstrating the internal consistency of the chosen critical exponents and validating the applicability of the scaling relations. For the Gd-based iQC, the modified Arrott analysis using mean-field critical exponents does not yield a comparable field range exhibiting parallel linear isotherms, indicating a clear deviation from mean-field behavior. As demonstrated in the main text, a self-consistent scaling analysis with non-mean-field critical exponents restores the linearity and confirms the anomalous critical behavior of this compound.

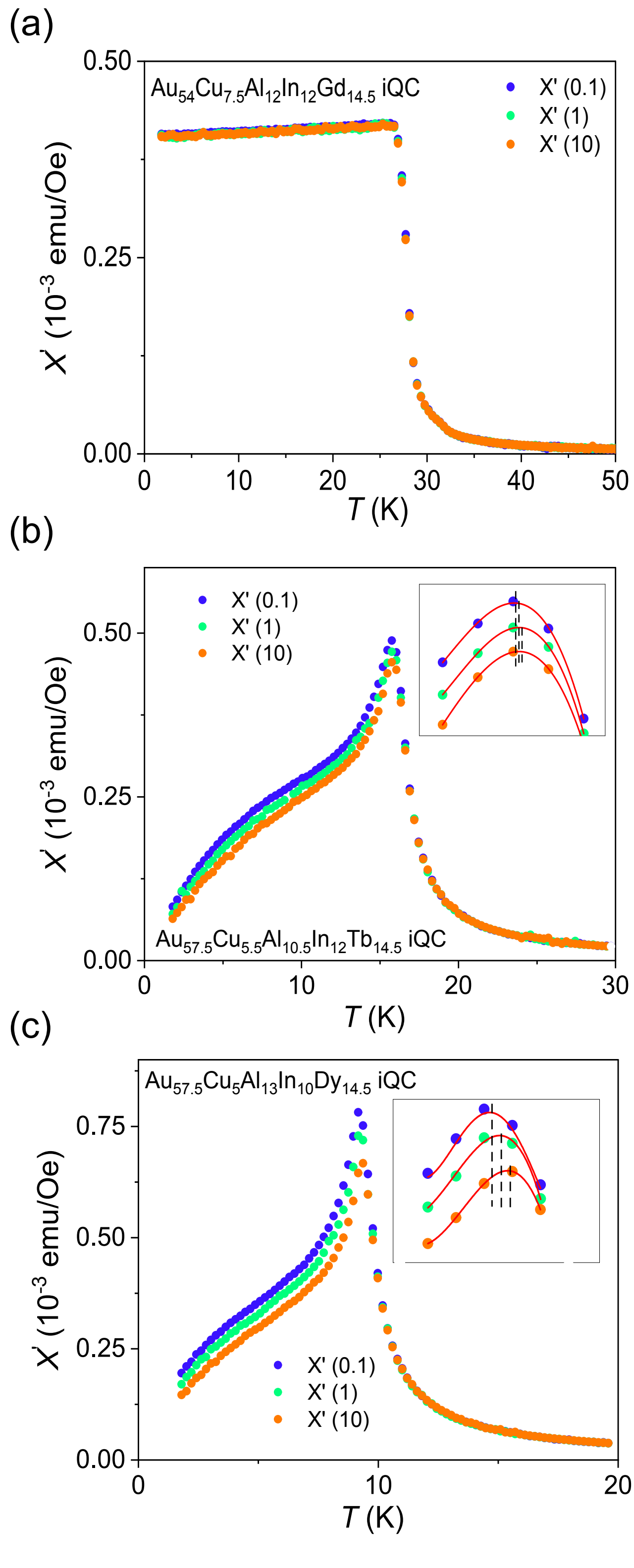

Figure S1. In-phase component of ac magnetic susceptibility (χ'_{ac}) for $Au_{54}Cu_{7.5}Al_{12}In_{12}Gd_{14.5}$, (b) $Au_{57.5}Cu_{5.5}Al_{10.5}In_{12}Tb_{14.5}$, and (c) $Au_{57.5}Cu_{5}Al_{13}In_{10}Dy_{14.5}$ under frequencies spanning two orders of magnitude from 0.1 to 10 Hz.

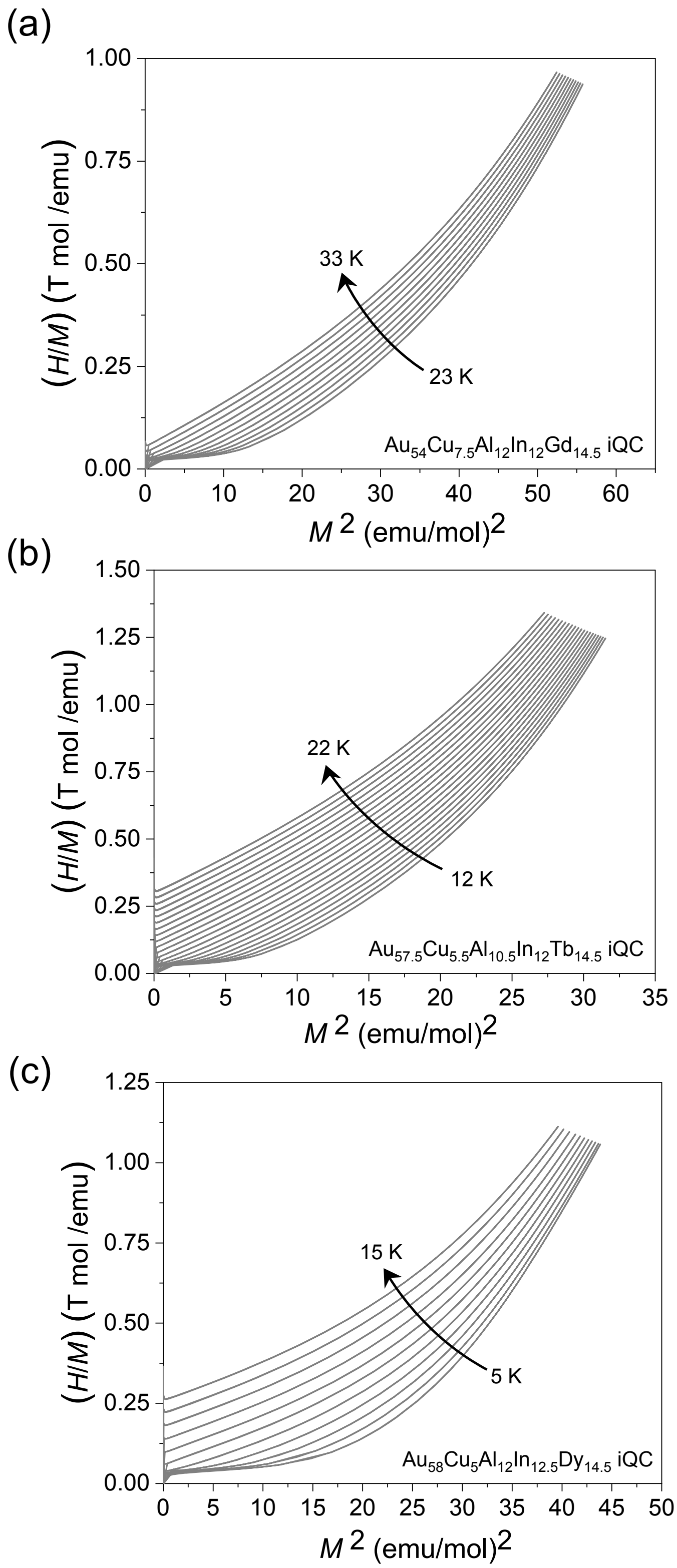

Figure S2. Arrott plots (*H/M* versus M^2) for (a) $Au_{54}Cu_{7.5}Al_{12}In_{12}Gd_{14.5}$, (b) $Au_{57.5}Cu_{5.5}Al_{10.5}In_{12}Tb_{14.5}$, and (c) $Au_{57.5}Cu_5Al_{13}In_{10}Dy_{14.5}$ iQCs in the vicinity of their respective Curie temperatures (*T*c).

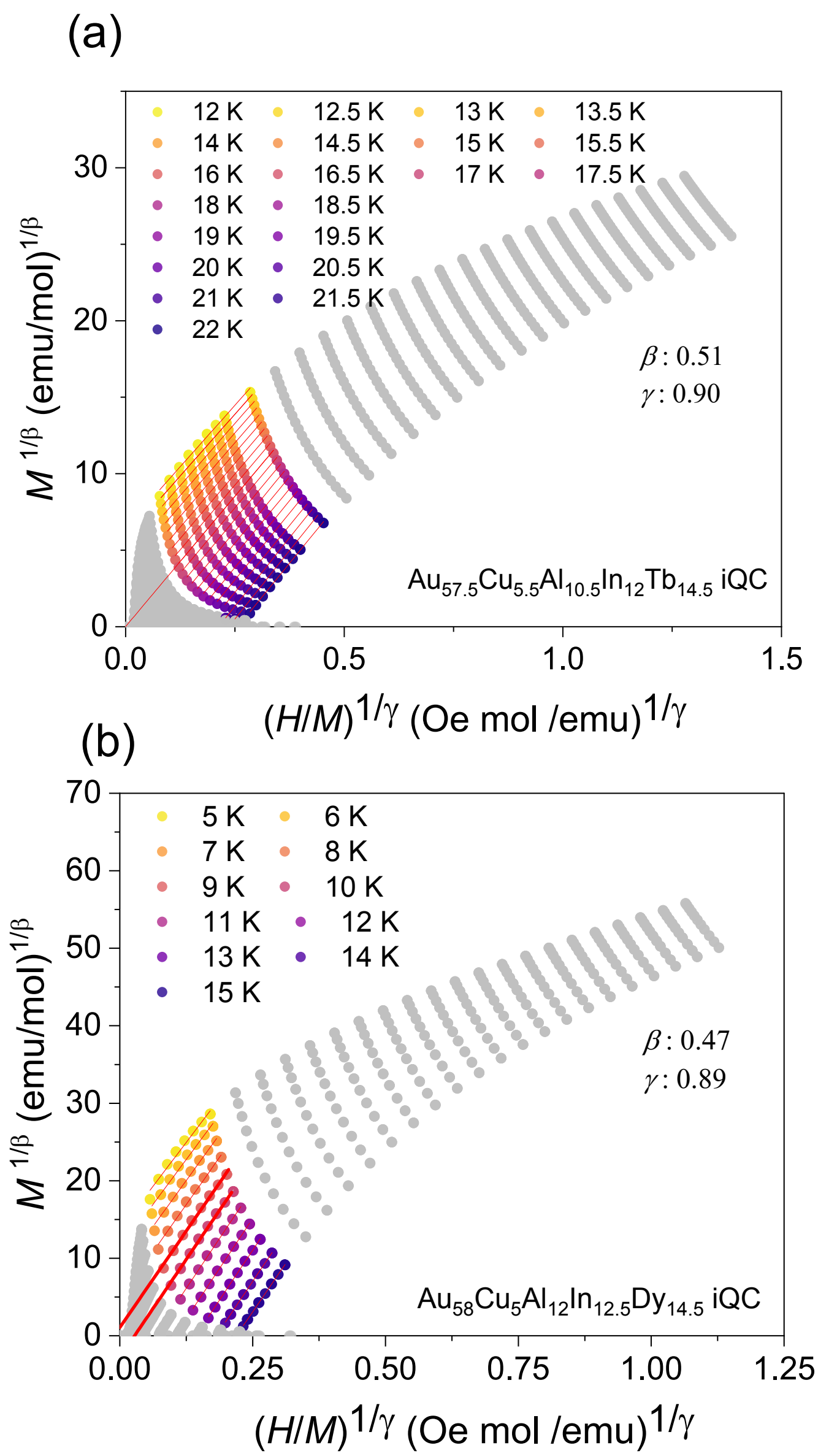

Figure S3. Modified Arrott isotherms ($M^{1/\beta}$ versus $H/M^{1/\gamma}$) for (a) $Au_{57.5}Cu_{5.5}Al_{10.5}In_{12}Tb_{14.5}$ and (b) $Au_{57.5}Cu_5Al_{13}In_{10}Dy_{14.5}$ iQCs. Nearly parallel linear fits are observed in the coloured regions of the isotherms, corresponding to magnetic fields in the range 0.4 T < H < 2.5 T.
